# Distress, multimorbidity, and complex multimorbidity among Chinese and Korean American older adults

**DOI:** 10.1371/journal.pone.0297035

**Published:** 2024-01-31

**Authors:** Hannah Oh, Brittany N. Morey, Yuxi Shi, Sunmin Lee

**Affiliations:** 1 Department of Public Health Sciences, Interdisciplinary Program in Precision Public Health, Graduate School of Korea University, Seoul, Republic of Korea; 2 Department of Health Policy and Management, College of Health Sciences, Korea University, Seoul, Republic of Korea; 3 Department of Medicine, School of Medicine, University of California, Irvine, Irvine, California, United States of America; 4 Department of Health, Society and Behavior, Program in Public Health, University of California, Irvine, Irvine, California, United States of America; Rey Juan Carlos University: Universidad Rey Juan Carlos, SPAIN

## Abstract

**Background:**

Studies suggest that distress is associated with various health conditions such as hypertension, asthma, diabetes, and coronary heart disease. However, only few studies focused on Asian Americans and little is known about the association with multiple comorbidity.

**Methods:**

We conducted a cross-sectional analysis among 400 Chinese and Korean American participants (aged 50–75 years) of the STOP CRC randomized controlled trial. Perceived distress was assessed using the distress thermometer scale (range 0–10). Disease diagnosis was self-reported by the participants. Multimorbidity (MM) was defined as having ≥2 chronic conditions. Complex multimorbidity (CMM) was defined as having ≥3 of the following body system disorders: circulation disorder, endocrine-metabolic disorder, cancer, anxiety or depression, breathing problem, and other health problems. We performed logistic regression for CMM and Poisson regression with robust error variance for MM to estimate associations with distress, adjusting for potential confounders.

**Results:**

The mean age was 58.4 years and mean distress score was 3.65. One-unit increase in distress score was associated with a 1.22-fold increase in the odds of having CMM (95% CI: 1.04–1.42). The magnitude of association slightly increased after additional adjustment for socioeconomic factors and health insurance status (OR: 1.29; 95% CI: 1.10–1.52). Higher distress score was positively associated with MM but the association was only marginally significant (PR: 1.04; 95% CI: 0.99–1.10), adjusting for socioeconomic factors and health insurance status.

**Conclusion:**

Our data suggest that higher perceived distress may be associated with simultaneous dysfunction of multiple distinct body systems among Chinese and Korean American older adults.

## Introduction

Distress refers to a spectrum of negative emotional experience, ranging from normal feelings of vulnerability, sadness, and fear to more severe problems such as anxiety, panic, and depression [1–3]. Distress is an important, yet overlooked, public health problem in Asian Americans. Studies reported that major sources of distress in Asian Americans are perceived racial discrimination and accultrative stress [4]. Discrimination and violence against Asians have particularly heightened since the COVID-19 pandemic [5,6]. The prevalence of Asian Americans experiencing distress also dramatically increased during the post-pandemic period [7]. Despite the growing concerns, Asian Americans are less likely to seek heath care services for mental health conditions compared with the general U.S. population [8,9], resulting in under-reported prevalence and delayed treatment. In some traditional Asian cultures, mental health problems are considered as shameful and a failure of emotional self-control [10,11]. Such mental health stigma, as well as language barrier and limited access to reliable information and resources, may discourage Asian Americans from seeking mental health care [11]. Therefore, to prevent the potential harms, it is important to fully understand the negative health effects of distress in Asian Americans.

Studies have shown that distress is associated with various health conditions such as sleep disturbance [12], asthma [13], and high blood pressure [14]. Distress can also promote the adoption of unhealthy behaviors [15,16], such as smoking [17], heavy alcohol drinking [18], and poor diet [19], all of which are important risk factors for various chronic diseases [20,21]. In prospective studies, distress was associated with increased risks of coronary heart disease [22], type 2 diabetes [23–25], and all-cause mortality [26]. However, only few studies focused on Asian Americans, whom may have different sources of distress and varying degree of severity and chronicity of conditions compared with the general U.S. population. Further, while distress may have systemic effects (e.g., chronic inflammation [27], DNA damage [28], microbiome imbalance [29]) that can simultaneously influence multiple organs of our body, little is known about the association of distress with multiple comorbidity. Multiple comorbidity requires the integrated care, often from multiple specialists. Racial and ethnic minorities are more likely to experience unmet medical needs for multiple comorbidity due to language barrier, lack of health insurance, and limited access to health care services [30]. Multiple comorbidity also contributes to poorer health outcomes, reduced quality of life [31], and higher healthcare cost [32], and thus more efforts should focus on the prevention by effectively targeting risk factors.

In this study, we examined the association between distress and multiple comorbidity in Chinese and Korean American older adults (aged 50–75 years), two of the largest Asian American subgroups in the US. While most studies focused on the association with a single disease, we investigated the association with multiple comorbidity using two different measures, multimorbidity (MM) and complex multimorbidity (CMM). MM indicates the co-occurrence of two or more chronic conditions. While MM is a simple measure based on the number of diseases within an individual, CMM indicates the co-occurrence of three or more chronic conditions affecting three or more different body systems [33] that require cares from different specialists. CMM may also indicate more severe health conditions and contribute to lower quality of life. CMM may provide additional information beyond MM by reflecting relevant pathogenic pathways and identifying the high-risk population with higher healthcare needs. Further, because sociodemographic factors, acculturation, sleep disorders, and health insurance status can influence individual’s response to distress, access to care, and susceptibility to chronic conditions, we also examined whether the associations are modified by these variables.

## Methods

### Study population

This analysis used the baseline data from 400 Chinese and Korean American participants (200 Chinese and 200 Korean Americans) of a randomized controlled trial called Screening To Prevent ColoRectal Cancer (STOP CRC) among At-Risk Asian American Primary Care Patients. Details of the study are described elsewhere [34]. Study participants were between the ages of 50 and 75 years, living in the Baltimore-Washington DC Metropolitan Area, and they were recruited from primary care physicians’ clinics in Maryland and Northern Virginia. The baseline survey data were collected from August 2018 to June 2020. For the current analysis, data were accessed from January to October 2022. Participants completed the survey either in-person or by phone in their preferred language (Mandarin, Korean, or English) after signing written informed consent forms. Eighty-nine percent of participants completed a self-administered questionnaire in-person; while 11% of participants completed a research assistant-led phone survey because of the COVID-19 outbreak in March 2020. In sensitivity analysis, we compared the results after excluding participants who responded via phone survey (n = 45; results are presented in S5 and S6 Tables). This study was approved by the Institutional Review Boards of the University of Maryland, College Park and the University of California, Irvine.

### Multimorbidity

Our dependent variables of interest were multimorbidity (MM) and complex multimorbidity (CMM) [35]. Participants were asked if they had ever been told by a doctor in the past year that they had following 10 health problems or conditions: (1) high blood pressure, (2) high cholesterol, (3) heart attack or any other heart disease, (4) cancer, (5) stroke, (6) diabetes, (7) obesity, (8) anxiety or depression, (9) breathing problem such as asthma or emphysema, and (10) any other health problems. Using this information, we created two binary variables, MM and CMM. MM was defined as having two or more of the 10 chronic conditions. CMM [35,36] was defined as having three or more of the following body system disorders: (1) endocrine-metabolic disorder (diabetes, obesity, or high cholesterol), (2) circulation disorder (high blood pressure, stroke, heart attack or any other heart disease), (3) cancer, (4) anxiety or depression, (5) breathing problem, and (6) other health problems. In sensitivity analysis, we re-defined CMM by excluding “other health problems” from the list of body system disorders (results are presented in S4 Table).

### Perceived distress

Distress is a subjective measure of perceived stress [2]. Level of overall perceived distress was assessed using the distress thermometer, a 0–10 visual analogue scale vertically oriented in the form of a thermometer (0 at the bottom indicating “no distress” and 10 at the top indicating “extreme distress”) [37]. Participants were asked to circle the number that best described how much distress they had been experiencing in the past week including the day of interview. Distress was used as a continuous variable ranging from 0 to 10, with higher scores indicating greater distress. In secondary analysis, we also categorized distress into 3 groups (low: ≤2; moderate: 3–5; high: ≥6) and compared across categories (results from secondary analyses are presented in S3 Table). The distress thermometer was derived from the National Comprehensive Cancer Network Distress Thermometer and Problem List (DTPL) and has been validly used in many psycho-oncological and non-oncological research settings across different cultures [37–39].

### Covariates

Sociodemographic characteristics including age, sex, Asian subgroup, marital status, education, household income, employment status, and health insurance status were self-reported at baseline. Age was used as a continuous variable in years. Sex was categorized as male and female. Asian subgroup was classified as Chinese and Korean. Education was grouped into three categories: high school/GED or less, business/vocational school/some college/college graduate, and some graduate/professional school. We categorized household income into three groups: <$40,000, $40,000-$99,999, and ≥ $100,000. Marital status was used as a binary variable: married/cohabiting (including married and living as married) and not currently married (including never married, widowed, divorced, and separated). Employment status was calssified as full-time, part-time, and not employed. Health insurance status was categorized as private health insurance, Medicare/Medicaid, and no health insurance. Sleep characteristics (sleep disturbance, sleep apnea, and sleep duration) were self-reported at baseline [34] and evaluated as effect modifiers in the analysis. Sleep disturbance was assessed using the adult 8-item version of the National Institutes of Health Patient Reported Outcomes Measurement Information System [40] and categorized into a binary variable (none to slight, mild/moderate/severe). Sleep apnea was assessed using the modified Berlin sleep apnea score [41] and categorized as low risk and high risk. Sleep duration was categorized as <6 and ≥6 hours.

### Statistical analysis

First, we conducted a descriptive analysis for the overall sample and after stratification by the level of CMM. Means and standard errors were calculated for continuous variables. Frequencies and percentages were reported for categorical variables. To compare the differences between subgroups, two sample t-tests were conducted for continuous variables and chi-square tests for categorical variables. Second, we used logistic regression models to estimate odds ratio (OR) and 95% confidence interval (CI) for the association between distress and CMM. For MM, we used Poission regression models with a robust error variance to estimate prevalence ratio (PR) and 95% confidene interval (CI) because MM was a common outcome with the prevalence of 38.3% in our study population. In a cross-sectional analysis with a common binary outcome (e.g., prevalence >10%), logistic regression can result in overestimated associations and Poisson regression models with a robust error variance may provide a better alternative [42,43]. For CMM, we also performed Poission regression models with a robust error variance in the sensitivity analysis (results are presented in S2 Table). For each outcome, multivariable models included the following variables: Model 1 included age; Model 2 added demographic factors (sex, Asian subgroup, marital status) to the Model 1; Model 3 added socioeconomic factors (education, household income, and employment status) to the Model 2; Model 4 added health insurance status to the Model 3. Then, we examined effect modification by age (< vs. ≥ mean age of 58 years), sex (male vs. female), Asian subgroup (Chinese vs. Korean Americans), socioeconomic factors (i.e., education, household income), acculturation levels (i.e., years in the U.S., self-rated English proficiency, self-rated accultration), health insurance status (with vs. without), and sleep patterns (i.e., sleep apnea, sleep disturbance, sleep duration). We also tested for interaction using the Wald test for interaction terms in the fully-adjusted models (stratified results with p-interaction<0.1 are presented in Table 5 and others in S7 Table). All statistical analyses were computed using Stata version 14.2.

## Results

### Study population

Table 1 displays the characteristics of study participants. Among 400 participants, the mean age was 58.4 years and 52.8% were female. The mean distress score was 3.65 and the prevalence of MM and CMM were 38.3% and 8.3%, respectively. Compared to participants without CMM, participants with CMM were more likley to be older (mean: 61.97 vs. 58.07 years) and have higher mean distress score (4.55 vs. 3.57). Participants with CMM were also less likely to be working full-time (30.3% vs. 60.2%) and having private health insurance (42.4% vs. 62.4%).

**Table 1:** Characteristics of study participants (n = 400).

	Total	Complex multimorbidity		p-value
		Absent	Present	
	n = 400 (100%)	n = 367 (91.8%)	n = 33 (8.3%)	
**Distress, mean (SE) (range: 0–10)**	3.65 (0.12)	3.57 (0.12)	4.55 (0.46)	0.03
**Age, mean (SE)**	58.39 (0.32)	58.07 (0.32)	61.97 (1.20)	0.001
**Sex, n (%)**				
Female	211 (52.8)	189 (51.5)	22 (66.7)	0.09
Male	189 (47.3)	178 (48.5)	11 (33.3)	
**Asian subgroup, n (%)**				
Chinese	200 (50.0)	187 (51.0)	13 (39.4)	0.20
Korean	200 (50.0)	180 (49.1)	20 (60.6)	
**Marital status, n (%)**				
Not currently married	59 (14.8)	56 (15.3)	3 (9.1)	0.34
Married/cohabiting	341 (85.3)	311 (84.7)	30 (90.9)	
**Education, n (%)**				
High school/GED or less	134 (33.5)	120 (32.7)	14 (42.4)	0.10
Business/vocational school/some college/college graduate	169 (42.3)	153 (41.7)	16 (48.5)	
Some graduate/professional school	97 (24.3)	94 (25.6)	3 (9.1)	
**Household income, n (%)**				
<$40,000	126 (31.5)	113 (30.8)	13 (39.4)	0.59
$40,000–99,999	166 (41.5)	154 (42.0)	12 (36.4)	
**≥**$100,000	108 (27.0)	100 (27.3)	8 (24.2)	
**Employment status, n (%)**				
Working full-time	231 (57.8)	221 (60.2)	10 (30.3)	0.001
Working part-time	84 (21.0)	76 (20.7)	8 (24.2)	
Not currently working	85 (21.3)	70 (19.1)	15 (45.5)	
**Health insurance status, n (%)**				
Private health insurance	243 (60.8)	229 (62.4)	14 (42.4)	0.04
Medicare/Medicaid	74 (18.5)	63 (17.2)	11 (33.3)	
No health insurance	83 (20.8)	75 (20.4)	8 (24.2)	

*Note*: SE = standard error.

Complex multimorbidity was defined as the coexistence of 3 or more of the following body system disorders: (1) circulation disorder (high blood pressure, stroke, heart attack or any other heart disease), (2) endocrine-metabolic disorder (diabetes, obesity, or high cholesterol), (3) cancer, (4) anxiety or depression, (5) breathing problem, and (6) any other health problems.

Table 2 presents the prevalence of individual chronic conditions and body system disorders in the study population. Among the 10 chronic conditions assessed in the study, high cholesterol (41.5%) was the most prevalent condition, followed by high blood pressure (34.0%) and diabetes (20.0%). Endocrine-metabolic disorder (51.0%) and circulation disorder (35.5%) were the most prevalent body system disorders. Although the prevalence of most chronic conditions were higher in Korean American participants, the overall disease profile was similar between Korean and Chinee American participants (S1 Table).

**Table 2:** Prevalence of individual chronic conditions and body system disorders in the study population (n = 400).

	Frequency	%
**Individual chronic condition** ^**a**^		
High blood pressure	136	34.0
High cholesterol	166	41.5
Heart attack or any other heart disease	24	6.0
Cancer	10	2.5
Stroke	7	1.8
Diabetes	80	20.0
Obesity	57	14.3
Anxiety or depression	23	5.8
Breathing problem	18	4.5
Any other health problems	47	11.8
**Body system disorder** ^**b**^		
Endocrine-metabolic disorder	204	51.0
Circulation disorder	142	35.5
Cancer	10	2.5
Anxiety or depression	23	5.8
Breathing problem	18	4.5
Other health problems	47	11.8

^a, b^ Prevalence was based on participants’ multiple choices.

^b^ Body system was categorized as follows: (1) circulation disorder (high blood pressure, stroke, heart attack or any other heart disease) (2) endocrine-metabolic disorder (diabetes, obesity, high cholesterol) (3) cancer (4) anxiety or depression (5) breathing problem (6) any other health problems.

### Association between distress and complex multimorbidity

Table 3 shows ORs and 95% CIs for the association between distress and CMM. Distress score was positively associated with CMM across all models. In Model 1, one-unit increase in distress score was associated with a 1.22-fold increase in the odds of having CMM (95% CI: 1.04–1.42). The magnitude of association slightly increased after additional adjustment for demographic and socioeconomic factors (Model 3: OR: 1.29; 95% CI: 1.09–1.51). No further change in the association was observed after further adjustment for health insurance status (Model 4: OR: 1.29; 95% CI: 1.10–1.52). When Poisson regression models with a robust error variance was performed, we observed similar results (S2 Table). In the fully-adjusted model, one-unit increase in distress score was associated with a 1.24-fold increase in the prevalence of CMM (95% CI: 1.09–1.41). When we analyzed using a categorical variable of distress, distress scores of 3–5 (PR: 1.84; 95% CI: 0.80–4.25) and ≥6 (PR: 3.17; 95% CI: 1.35–7.41), compared with score ≤2, were both positively associated with CMM but the association was statistically significant for distress score of ≥6 only (S3 Table). We observed similar results after excluding “other health problems” from the definition of CMM (S4 Table) and after excluding participants who responded via phone survey (S5 and S6 Tables).

**Table 3:** Odds ratio (OR) and 95% confidence interval (CI) for the association between distress and complex multimorbidity (n = 400).

	Complex multimorbidity (CMM)^a^			
	OR (95% CI)^b^			
	Model 1^c^	Model 2^d^	Model 3^e^	Model 4^f^
**Distress score**				
Per 1-unit increase	1.22 (1.04–1.42)	1.22 (1.05–1.43)	1.29 (1.09–1.51)	1.29 (1.10–1.52)
**Age**				
Per 1-year increase	1.11 (1.04–1.17)	1.11 (1.05–1.18)	1.09 (1.01–1.16)	1.08 (1.00–1.17)
**Sex**				
Male		1.00 (Ref)	1.00 (Ref)	1.00 (Ref)
Female		2.26 (1.03–4.94)	1.47 (0.62–3.46)	1.51 (0.64–3.60)
**Asian subgroup**				
Korean		1.00 (Ref)	1.00 (Ref)	1.00 (Ref)
Chinese		0.86 (0.40–1.85)	0.87 (0.38–2.00)	0.85 (0.36–1.99)
**Marital status**				
Married/cohabiting		1.00 (Ref)	1.00 (Ref)	1.00 (Ref)
Not currently married		0.32 (0.09–1.19)	0.40 (0.10–1.56)	0.42 (0.11–1.60)
**Education**				
High school/GED or less			1.00 (Ref)	1.00 (Ref)
Business/vocational school/some college/college graduate			0.83 (0.35–1.99)	0.86 (0.36–2.05)
Some graduate/professional school			0.25 (0.05–1.13)	0.25 (0.06–1.15)
**Household income**				
<$40,000			1.00 (Ref)	1.00 (Ref)
$40,000–99,999			0.96 (0.36–2.53)	0.94 (0.35–2.53)
**≥**$100,000			2.86 (0.84–9.72)	3.55 (0.97–12.92)
**Employment status**				
Working full time			1.00 (Ref)	1.00 (Ref)
Working part time			2.56 (0.87–7.52)	2.62 (0.88–7.80)
Not currently working			3.40 (1.21–9.53)	3.32 (1.17–9.42)
**Health insurance status**				
Private health insurance				1.00 (Ref)
Medicare/Medicaid				1.41 (0.46–4.33)
No health insurance				1.85 (0.65–5.27)

^a^ Complex multimorbidity was defined as having three or more of the following body system disorders: (1) endocrine-metabolic disorder (diabetes, obesity, or high cholesterol), (2) circulation disorder (high blood pressure, stroke, heart attack or any other heart disease), (3) cancer, (4) anxiety or depression, (5) breathing problem, and (6) any other health problems.

^b^ Odds ratio and 95% confidence intervals were estimated from the logistic regression models.

^c^ Model 1 adjusted for age.

^d^ Model 2 adjusted for age, sex, Asian subgroup, and marital status.

^e^ Model 3 adjusted for age, sex, Asian subgroup, marital status, education, household income, and employment status.

^f^ Model 4 adjusted for age, sex, Asian subgroup, marital status, education, household income, employment status, and health insurance status.

Among the covariates, older age was associated with higher odds of CMM across all models (Table 3). Being female was also positively associated with CMM, but the association was statistically significant only in the Model 2 (OR: 2.26; 95% CI: 1.03–4.94). Not currently working compared to working full time was associated with higher odds of CMM in both Model 3 (OR: 3.40; 95% CI: 1.21–9.53) and Model 4 (OR: 3.32; 95% CI: 1.17–9.42).

### Association between distress and multimorbidity

Table 4 presents the association between distress and MM. Higher distress score was positively associated with MM but the association was only marginally significant (Model 4: PR: 1.04; 95% CI: 0.99–1.10). Similar to the results for CMM in Table 3, age was positively associated with MM across all models. Compared to participants who had less than high school education, those who attended graduate or professional school had lower prevalene of MM (Model 4: PR: 0.65; 95% CI: 0.43–0.99). Compared to those employed full-time, those who were not working had higher prevalence of MM (Model 4: PR: 1.42; 95% CI: 1.02–1.98).

**Table 4:** Prevalence ratio (PR) and 95% confidence interval (CI) for the association between distress and multimorbidity (n = 400).

	Multimorbidity (MM)^a^			
	PR (95% CI)^b^			
	Model 1^c^	Model 2^d^	Model 3^e^	Model 4^f^
**Distress score**				
Per 1-unit increase	1.03 (0.98–1.08)	1.03 (0.98–1.08)	1.04 (0.99–1.10)	1.04 (0.99–1.10)
**Age**				
Per 1-year increase	1.04 (1.03–1.06)	1.04 (1.02–1.06)	1.03 (1.01–1.06)	1.03 (1.01–1.06)
**Sex**				
Male		1.00 (Ref)	1.00 (Ref)	1.00 (Ref)
Female		1.06 (0.83–1.37)	0.93 (0.71–1.22)	0.93 (0.71–1.22)
**Asian subgroup**				
Korean		1.00 (Ref)	1.00 (Ref)	1.00 (Ref)
Chinese		0.84 (0.64–1.08)	0.86 (0.66–1.13)	0.85 (0.64–1.12)
**Marital status**				
Married/cohabiting		1.00 (Ref)	1.00 (Ref)	1.00 (Ref)
Not currently married		0.98 (0.69–1.38)	1.03 (0.73–1.45)	1.03 (0.73–1.46)
**Education**				
High school/GED or less			1.00 (Ref)	1.00 (Ref)
Business/vocational school/some college/college graduate			0.92 (0.70–1.22)	0.92 (0.70–1.21)
Some graduate/professional school			0.66 (0.43–1.01)	0.65 (0.43–0.99)
**Household income**				
<$40,000			1.00 (Ref)	1.00 (Ref)
$40,000–99,999			1.01 (0.75–1.36)	0.99 (0.74–1.34)
**≥**$100,000			1.25 (0.86–1.82)	1.26 (0.86–1.85)
**Employment status**				
Working full time			1.00 (Ref)	1.00 (Ref)
Working part time			1.42 (1.03–1.96)	1.43 (1.03–1.97)
Not currently working			1.42 (1.02–1.96)	1.42 (1.02–1.98)
**Health insurance status**				
Private health insurance				1.00 (Ref)
Medicare/Medicaid				0.92 (0.64–1.33)
No health insurance				1.08 (0.79–1.48)

^a^ Multimorbidity was defined as having two or more of the following individual chronic conditions: (1) high blood pressure, (2) high cholesterol, (3) heart attack or any other heart disease, (4) cancer, (5) stroke, (6) diabetes, (7) obesity, (8) anxiety or depression, (9) breathing problem, and (10) any other health problems.

^b^ Prevalence ratio and 95% confidence intervals were estimated from the Poisson regression models with a robust error variance.

^c^ Model 1 adjusted for age.

^d^ Model 2 adjusted for age, sex, Asian subgroup, and marital status.

^e^ Model 3 adjusted for age, sex, Asian subgroup, marital status, education, household income, and employment status.

^f^ Model 4 adjusted for age, sex, Asian subgroup, marital status, education, household income, employment status, and health insurance status.

### Stratified analyses

When we stratified the analyses by potential effect modifiers, the positive association with CMM was slightly more pronounced in female parcitipants (p-interaction = 0.08) and the positive association with MM was restricted to younger participants (p-interaction = 0.09; Table 5). However, none of the interactions was statistically significant at alpha 0.05 (Table 5 and S7 Table).

**Table 5:** Associations of distress with complex multimorbidity (CMM) and multimorbidity (MM), stratified by age, sex, and health insurance status (n = 400).

	Complex multimorbidity (CMM)			Multimorbidity (MM)		
	N	OR (95% CI)^a^	p-int ^b^	N	PR (95% CI)^c^	p-int ^b^
**Age**						
<58 years	197	1.17 (0.88–1.57)	0.62	197	1.11 (1.03–1.21)	0.09
≥58 years	203	1.31 (1.07–1.61)		203	1.01 (0.95–1.07)	
**Sex**						
Male	189	1.09 (0.81–1.45)	0.08	189	1.05 (0.98–1.13)	0.85
Female	211	1.45 (1.16–1.82)		211	1.04 (0.97–1.11)	
**Health insurance status**						
Without health insurance	83	1.66 (1.04–2.65)	0.52	83	1.05 (0.95–1.16)	0.08
With health insurance	317	1.23 (1.02–1.49)		317	1.06 (1.00–1.12)	

^a^ Odds ratio (OR) and 95% confidence interval (CI) were estimated using logistic regression models adjusting for age, sex, Asian subgroup, marital status, education, household income, employment status, and health insurance status.

^b^ p-interaction was estimated using Wald test for interaction terms

^c^ Prevalence ratio (PR) and 95% confidence interval (CI) were estimated using Poisson regression models with a robust error variance, adjusting for age, sex, Asian subgroup, marital status, education, household income, employment status, and health insurance status.

## Discussion

To the best of our knowledge, this study is the first to examine the association between distress and multiple comorbidity among Chinese and Korean Americans. In this cross-sectional analysis, we observed that higher perceived distress was associated with higher prevalence of CMM, indicated by the presence of 3 or more affected body systems. The associations were persistent after adjustment for demographic characteristics, socioeconomic factors, and health insurance status. The positive direction of association was also observed between distress and MM but the association was not statistically significant. Our data suggest that higher perceived distress may be associated with simultaneous dysfunction of multiple distinct body systems in Chinese and Korean Americans.

Our finding of positive association between distress and CMM is consistent with the result from a previous study [44]. In a study of 238 patients from primary care clinics in Canada, higher level of distress was positively associated with cumulative illness rating scale (CIRS), the multiple comorbidity measure that accounted for affected organ system and disease severity (e.g., greater weights were given to more severe conditions) [44]. Similar to our finding, the association also persisted after the adjustment for socioeconomic status. Although low socioeconomic status such as unemployment may be a potential risk factor for CMM, these findings suggest that socioeconomic status is unlikely to fully explain the positive association we observed between distress and CMM. In addition, our analysis of categorical distress variable also showed a dose-response relationship with CMM, further supporting the positive association between distress and CMM.

However, among the studies that assessed multiple comorbidity based on a simple disease count [44–46], the results were mixed. In the studies from Northern India [45] and African Americans in the U.S. [46], distress was associated with a higher count of chronic conditions, while our study of Asian Americans and the previous study from Canada [44] did not observe a statistically significant association with the measure based on a simple disease count. The discrepancy in study findings may be due to the differences in study population (e.g., morbidity profile) and assessment method of multiple comorbidity. In the study from Northern India, the most prevalent condition among the study participants was anemia [45], showing a different morbidity profile compared with those observed in the U.S. and Canada. In our study, the most common body system disorder was endocrine-metabolic disorder, including diabetes, obesity, and high cholesterol, among both Chinese and Korean American participants. In the study from African Americans in the U.S., hypertension and arthritis were the most common health conditions [46]. When assssing multiple comorbidity, different studies also used different lists of diseases. For example, the study from Canada [44] used a list of 14 different domains of diseases that were classified by affected body system (e.g., cardiac, respiratory, renal) while other studies used a list of individual chronic conditions (e.g., obesity, diabetes, hypertension) [45,46].

In our study, we observed a statistically significant association of distress with CMM but not with MM. It is likely that CMM is a better measure in differentiating the high-risk population than MM, as the prevalence of MM is already high in our study population. Further, by grouping diseases by the biologically relevant body system, CMM is likely a more reliable measure of multiple comorbidity. Misclassification is less likely to occur when assessing groups of closely-related diseases compared with when assessing individual diseases.

There are several potential mechanisms that may explain the adverse effects of distress on multiple comorbidity. First, distress may simultaneously increase the risks of multiple chronic conditions through biological effects that influence multiple organs of our body. Distress can disrupt the hypothalamic-pituitary-adrenal axis [47], leading to increased secretion of cortisol [48]. Elevated levels of cortisol are associated with sleep disturbance [49], immune suppression [50], appetite dysregulation [51], and chronic inflammation [52]. These changes may lead to altered glucose metabolism [53] and increase the risks of different chronic diseases such as type 2 diabetes [23–25], cardiovascular disease [54], and certain cancers [55]. Sleep disturbance and poor sleep quality are associated with weight gain [56], high blood pressure [57], and mortality [58–60]. Distress can also disrupt the balance of gut microbiome by releasing stress hormones and creating pro-inflammatory environment [29]. Some gut bacteria can release toxins that have detrimental effects on cardiometabolic health [61,62]. Second, distress can also indirectly increase the risks of multiple chronic conditions by promoting the adoption of unhealthy behaviors [15,16]. Cravings for unhealthy foods, heavy alcohol drinking, smoking, and substance abuse are often used as coping methods for distress. Lastly, distress may make the management of disease more difficult, resulting in development of disease-related complications and poor health outcomes. Studies have shown that individuals experiencing distress are more likely to show a lower adherence to treatment [63], leading to worsening of disease severity and outcomes.

This study has important strengths. By using a subjective measure of distress, we were able to include less severe conditions that may not have been clinically diagnosed among Asian Americans with low utilization of mental health care. The single-item distress scale may also better reflect the individual’s overall perception of stressors. In our analysis, we also showed the robustness of our study results. The association between distress and CMM persisted after the adjustment for various demographic and socioeconomic factors.

We also acknowledge several limitations of this study. First, given the cross-sectional study design, the temporal relationship between distress and multiple comorbidity is unclear. Having diagnosed with several chronic conditions can also lead to development of distress [64]. Because the bidirectional relationship is possible, longitudinal studies are needed to confirm our results. Second, we used the self-reported data of physician-diagnosed diseases. The data may be subject to measurement error if study participants did not accurately remember the diagnosis. It is also possible that some health conditions may have been under-reported among individuals with infrequent clinic visit. Twenty-one percent of our study participants did not have health insurance, suggesting that these individuals are likely to have lower health care utilization compared with the general U.S. population. It is also possible that the COVID-19 pandemic reduced access to medical care, leading to underdiagnosis of mental and physical health conditions and thereby underestimation of the associations. However, in our study, only a small portion of data (11%, n = 45) were collected via phone survey during the pendemic, while the rest of data were collected via self-administered questionnaire before the pandemic. Although the accuracy of data may be different between the two methods, our sensitivity analysis confirmed that the results were similar when we restricted the analysis to the data collected via self-administered questionnaire. Lastly, our study population included Chinese and Korean Americans aged 50–75 years and thus our study results may not be generalizable to other racial populations or younger age groups with different severity of distress and susceptibility to chronic conditions.

In summary, we observed that higher perceived distress was associated with higher prevalence of multiple comorbidity measured by CMM among Chinese and Korean Americans. Our data provide additional insights into the potential risk factors for multiple comorbidity. Our data also highlight the importance of raising awareness on distress and related mental health problems and promoting utilization of mental health care among Asian American older adults.

## Supporting information

S1 Table Prevalence of individual chronic conditions and body system disorders in Korean and Chinese American participants (n = 400). (DOCX)

S2 Table Prevalence ratio (OR) and 95% confidence interval (CI) for the association between distress and complex multimorbidity (CMM), estimated from Poisson regression models with a robust error variance (n = 400). (DOCX)

S3 Table The associations of categorical distress score with complex multimorbidity (CMM) and multimorbidity (MM) (n = 400). (DOCX)

S4 Table Odds ratio (OR) and 95% confidence interval (CI) for the association between distress and complex multimorbidity (CMM), after excluding “other health problems” from the CMM definition (n = 400). (DOCX)

S5 Table Odds ratio (OR) and 95% confidence interval (CI) for the association between distress and complex multimorbidity (CMM), after excluding participants responded via phone survey (n = 355). (DOCX)

S6 Table Prevalence ratio (PR) and 95% confidence interval (CI) for the association between distress and multimorbidity (MM), after excluding participants responded via phone survey (n = 355). (DOCX)

S7 Table Associations of distress with complex multimorbidity (CMM) and multimorbidity (MM), stratified by Asian subgroup, socioeconomic factors, acculturation level, and sleep patterns (n = 400). (DOCX)
