# Supplementary material for: Distress, multimorbidity, and complex multimorbidity among Chinese and Korean American older adults
Source: PLoS One. 2024 Jan 31;19(1):e0297035. doi: 10.1371/journal.pone.0297035 (PMC10830023; doi:10.1371/journal.pone.0297035)
Supplement: S1 Table — (DOCX) [file pone.0297035.s001.docx]

**S1 Table. Prevalence of individual chronic conditions and body system disorders in Korean and Chinese American participants (n=400)**

|  | **Korean**  **(n=200)** | | **Chinese**  **(n=200)** | |
| --- | --- | --- | --- | --- |
|  | **Frequency** | **%** | **Frequency** | **%** |
| **Individual chronic condition** ^a^ |  |  |  |  |
| High blood pressure | 87 | 43.5 | 49 | 24.5 |
| High cholesterol | 91 | 45.5 | 75 | 37.5 |
| Heart attack or any other heart disease | 13 | 6.5 | 11 | 5.5 |
| Cancer | 6 | 3.0 | 4 | 2.0 |
| Stroke | 6 | 3.0 | 1 | 0.5 |
| Diabetes | 52 | 26.0 | 28 | 14.0 |
| Anxiety or depression | 9 | 4.5 | 14 | 7.0 |
| Obesity | 33 | 16.5 | 24 | 12.0 |
| Breathing problem | 11 | 5.5 | 7 | 3.5 |
| Any other health problems | 23 | 11.5 | 24 | 12.0 |
| **Body system disorder** ^b^ |  |  |  |  |
| Circulation disorder | 88 | 44.0 | 54 | 27.0 |
| Cancer | 6 | 3.0 | 4 | 2.0 |
| Endocrine-metabolic disorder | 114 | 57.0 | 90 | 45.0 |
| Anxiety or depression | 9 | 4.5 | 14 | 7.0 |
| Breathing problem | 11 | 5.5 | 7 | 3.5 |
| Any other health problems | 23 | 11.5 | 24 | 12.0 |

^a, b^ Prevalence was based on participants’ multiple choices.

^b^ Body system was categorized as follows: (1) circulation disorder (high blood pressure, stroke, heart attack or any other heart disease) (2) endocrine-metabolic disorder (diabetes, obesity, high cholesterol) (3) cancer (4) anxiety or depression (5) breathing problem (6) any other health problems
