# Supplementary material for: Distress, multimorbidity, and complex multimorbidity among Chinese and Korean American older adults
Source: PLoS One. 2024 Jan 31;19(1):e0297035. doi: 10.1371/journal.pone.0297035 (PMC10830023; doi:10.1371/journal.pone.0297035)
Supplement: S2 Table — (DOCX) [file pone.0297035.s002.docx]

**S2 Table. Prevalence ratio (OR) and 95% confidence interval (CI) for the association between distress and complex multimorbidity (CMM), estimated from Poisson regression models with a robust error variance (n=400)**

|  | **Complex multimorbidity (CMM)** | | | |
| --- | --- | --- | --- | --- |
|  | **PR (95% CI)^a^** | | | |
|  | **Model 1^b^** | **Model 2^c^** | **Model 3^d^** | **Model 4^e^** |
| **Distress score** |  |  |  |  |
| Per 1-unit increase | 1.19 (1.03-1.37) | 1.19 (1.03-1.37) | 1.23 (1.08-1.40) | 1.24 (1.09-1.41) |
| **Age** |  |  |  |  |
| Per 1-year increase | 1.09 (1.04-1.14) | 1.10 (1.04-1.15) | 1.07 (1.02-1.13) | 1.07 (1.00-1.14) |
| **Sex** |  |  |  |  |
| Male |  | 1.00 (Ref) | 1.00 (Ref) | 1.00 (Ref) |
| Female |  | 2.01 (1.02-3.93) | 1.37 (0.63-3.00) | 1.41 (0.64-3.08) |
| **Asian subgroup** |  |  |  |  |
| Korean |  | 1.00 (Ref) | 1.00 (Ref) | 1.00 (Ref) |
| Chinese |  | 0.87 (0.45-1.67) | 0.89 (0.44-1.81) | 0.87 (0.42-1.78) |
| **Marital status** |  |  |  |  |
| Married/cohabiting |  | 1.00 (Ref) | 1.00 (Ref) | 1.00 (Ref) |
| Not currently married |  | 0.37 (0.13-1.04) | 0.45 (0.17-1.25) | 0.47 (0.17-1.28) |
| **Education** |  |  |  |  |
| High school/GED or less |  |  | 1.00 (Ref) | 1.00 (Ref) |
| Business/vocational school/some college/college graduate |  |  | 0.86 (0.43-1.72) | 0.88 (0.45-1.73) |
| Some graduate/professional school |  |  | 0.30 (0.09-1.03) | 0.30 (0.09-1.03) |
| **Household income** |  |  |  |  |
| <$40,000 |  |  | 1.00 (Ref) | 1.00 (Ref) |
| $40,000-99,999 |  |  | 0.98 (0.44-2.19) | 0.95 (0.42-2.18) |
| **≥**$100,000 |  |  | 2.42 (0.89-6.59) | 2.92 (0.95-8.94) |
| **Employment status** |  |  |  |  |
| Working full time |  |  | 1.00 (Ref) | 1.00 (Ref) |
| Working part time |  |  | 2.32 (0.88-6.13) | 2.35 (0.90-6.14) |
| Not currently working |  |  | 2.79 (1.18-6.59) | 2.74 (1.15-6.55) |
| **Health insurance status** |  |  |  |  |
| Private health insurance |  |  |  | 1.00 (Ref) |
| Medicare/Medicaid |  |  |  | 1.34 (0.51-3.52) |
| No health insurance |  |  |  | 1.73 (0.75-4.00) |

^a^Prevalence ratio and 95% confidence intervals were estimated from the Poisson regression models with a robust error variance.

^b^Model 1 adjusted for age.

^c^Model 2 adjusted for age, sex, Asian subgroup, and marital status.

^d^Model 3 adjusted for age, sex, Asian subgroup, marital status, education, household income, and employment status.

^e^Model 4 adjusted for age, sex, Asian subgroup, marital status, education, household income, employment status, and health insurance status.
