# Supplementary material for: Distress, multimorbidity, and complex multimorbidity among Chinese and Korean American older adults
Source: PLoS One. 2024 Jan 31;19(1):e0297035. doi: 10.1371/journal.pone.0297035 (PMC10830023; doi:10.1371/journal.pone.0297035)
Supplement: S3 Table — (DOCX) [file pone.0297035.s003.docx]

**S3 Table. The associations of categorical distress score with complex multimorbidity (CMM) and multimorbidity (MM) (n=400)**

|  | **Complex multimorbidity (CMM)** | | **Multimorbidity (MM)** |
| --- | --- | --- | --- |
|  | **Logistic regression** | **Poisson regression with robust error variance** | **Poisson regression with robust error variance** |
|  | **OR (95% CI)** | **PR (95% CI)** | **PR (95% CI)** |
| **Distress score** |  |  |  |
| Low (≤2) | 1.00 | 1.00 | 1.00 |
| Moderate (3-5) | 2.02 (0.76-5.37) | 1.84 (0.80-4.25) | 1.08 (0.82-1.42) |
| High (≥6) | 4.00 (1.39-11.5) | 3.17 (1.35-7.41) | 1.24 (0.89-1.71) |
| **Age** |  |  |  |
| Per 1-year increase | 1.08 (1.00-1.16) | 1.07 (1.00-1.13) | 1.03 (1.01-1.06) |
| **Sex** |  |  |  |
| Male | 1.00 | 1.00 | 1.00 |
| Female | 1.47 (0.61-3.52) | 1.37 (0.60-3.08) | 0.93 (0.71-1.22) |
| **Asian subgroup** |  |  |  |
| Korean | 1.00 | 1.00 | 1.00 |
| Chinese | 0.87 (0.37-2.03) | 0.88 (0.43-1.83) | 0.85 (0.64-1.12) |
| **Marital status** |  |  |  |
| Married/cohabit | 1.00 | 1.00 | 1.00 |
| Not currently married | 0.42 (0.11-1.62) | 0.47 (0.17-1.33) | 1.03 (0.73-1.46) |
| **Education** |  |  |  |
| High school/GED or less | 1.00 | 1.00 | 1.00 |
| Business/vocational school/some college/college graduate | 0.93 (0.39-2.22) | 0.95 (0.48-1.88) | 0.93 (0.71-1.22) |
| Attended graduate/professional school | 0.27 (0.06-1.23) | 0.31 (0.09-1.06) | 0.66 (0.43-1.01) |
| **Household income** |  |  |  |
| <$40,000 | 1.00 | 1.00 | 1.00 |
| $40,000-99,999 | 0.95 (0.35-2.55) | 0.97 (0.41-2.29) | 1.00 (0.74-1.35) |
| **≥**$100,000 | 3.33 (0.92-12.03) | 2.84 (0.94-8.60) | 1.25 (0.85-1.84) |
| **Employment status** |  |  |  |
| Working full time | 1.00 | 1.00 | 1.00 |
| Working part time | 2.44 (0.82-7.21) | 2.22 (0.83-5.95) | 1.41 (1.02-1.95) |
| Not currently working | 3.10 (1.10-8.75) | 2.63 (1.06-6.52) | 1.41 (1.01-1.96) |
| **Health insurance** |  |  |  |
| Private health insurance | 1.00 | 1.00 | 1.00 |
| Medicare/Medicaid | 1.45 (0.48-4.39) | 1.36 (0.52-3.52) | 0.93 (0.65-1.33) |
| No health insurance | 1.87 (0.66-5.31) | 1.76 (0.74-4.15) | 1.08 (0.79-1.48) |

All models adjusted for age, sex, Asian subgroup, marital status, education, household income, employment status, and health insurance status.
