# Supplementary material for: Distress, multimorbidity, and complex multimorbidity among Chinese and Korean American older adults
Source: PLoS One. 2024 Jan 31;19(1):e0297035. doi: 10.1371/journal.pone.0297035 (PMC10830023; doi:10.1371/journal.pone.0297035)
Supplement: S6 Table — (DOCX) [file pone.0297035.s006.docx]

**S6 Table. Prevalence ratio (PR) and 95% confidence interval (CI) for the association between distress and multimorbidity (MM) after excluding participants responded via phone survey (n=355)**

|  | **Multimorbidity (MM)^a^** | | | |
| --- | --- | --- | --- | --- |
|  | **PR (95% CI)^b^** | | | |
|  | **Model 1^c^** | **Model 2^d^** | **Model 3^e^** | **Model 4^f^** |
| **Distress score** |  |  |  |  |
| Per 1-unit increase | 1.03 (0.98-1.08) | 1.02 (0.97-1.08) | 1.04 (0.99-1.09) | 1.04 (0.99-1.09) |
| **Age** |  |  |  |  |
| Per 1-year increase | 1.05 (1.03-1.07) | 1.05 (1.02-1.07) | 1.04 (1.01-1.06) | 1.04 (1.02-1.07) |
| **Sex** |  |  |  |  |
| Male |  | 1.00 | 1.00 | 1.00 |
| Female |  | 1.10 (0.85-1.43) | 0.98 (0.74-1.30) | 0.96 (0.73-1.28) |
| **Asian subgroup** |  |  |  |  |
| Korean |  | 1.00 | 1.00 | 1.00 |
| Chinese |  | 0.92 (0.70-1.21) | 0.96 (0.72-1.27) | 0.93 (0.70-1.25) |
| **Marital status** |  |  |  |  |
| Married/cohabiting |  | 1.00 | 1.00 | 1.00 |
| Not currently married |  | 0.97 (0.69-1.38) | 1.03 (0.72-1.46) | 1.04 (0.73-1.48) |
| **Education** |  |  |  |  |
| High school/GED or less |  |  | 1.00 | 1.00 |
| Business/vocational school/some college/college graduate |  |  | 0.99 (0.75-1.32) | 0.97 (0.73-1.29) |
| Some graduate/professional school |  |  | 0.70 (0.45-1.09) | 0.68 (0.44-1.06) |
| **Household income** |  |  |  |  |
| <$40,000 |  |  | 1.00 | 1.00 |
| $40,000-99,999 |  |  | 1.02 (0.75-1.38) | 1.00 (0.74-1.36) |
| **≥**$100,000 |  |  | 1.21 (0.82-1.79) | 1.19 (0.80-1.78) |
| **Employment status** |  |  |  |  |
| Working full time |  |  | 1.00 | 1.00 |
| Working part time |  |  | 1.35 (0.97-1.88) | 1.37 (0.98-1.90) |
| Not currently working |  |  | 1.38 (0.98-1.94) | 1.41 (1.00-2.00) |
| **Health insurance status** |  |  |  |  |
| Private health insurance |  |  |  | 1.00 |
| Medicare/Medicaid |  |  |  | 0.85 (0.58-1.23) |
| No health insurance |  |  |  | 1.02 (0.74-1.41) |

^a^ Multimorbidity was defined as having two or more of the following individual chronic conditions: (1) high blood pressure, (2) high cholesterol, (3) heart attack or any other heart disease, (4) cancer, (5) stroke, (6) diabetes, (7) obesity, (8) anxiety or depression, (9) breathing problem, and (10) any other health problems.

^b^ Prevalence ratio and 95% confidence intervals were estimated from the Poisson regression models with a robust error variance.

^c^ Model 1 adjusted for age.

^d^ Model 2 adjusted for age, sex, Asian subgroup, and marital status.

^e^ Model 3 adjusted for age, sex, Asian subgroup, marital status, education, household income, and employment status.

^f^ Model 4 adjusted for age, sex, Asian subgroup, marital status, education, household income, employment status, and health insurance status.
