# Supplementary material for: Distress, multimorbidity, and complex multimorbidity among Chinese and Korean American older adults
Source: PLoS One. 2024 Jan 31;19(1):e0297035. doi: 10.1371/journal.pone.0297035 (PMC10830023; doi:10.1371/journal.pone.0297035)
Supplement: S7 Table — (DOCX) [file pone.0297035.s007.docx]

**S7 Table. Associations of distress with complex multimorbidity (CMM) and multimorbidity (MM), stratified by Asian subgroup, socioeconomic factors, acculturation level, and sleep patterns (n=400)**

|  | **Complex multimorbidity (CMM)** | | | **Multimorbidity (MM)** | | |
| --- | --- | --- | --- | --- | --- | --- |
|  | **N** | **OR (95% CI)^a^** | **p-int ^b^** | **N** | **PR (95% CI)^c^** | **p-int ^b^** |
| **Asian subgroup** |  |  |  |  |  |  |
| Chinese | 200 | 1.46 (1.08-1.97) | 0.54 | 200 | 1.08 (0.99-1.17) | 0.99 |
| Korean | 200 | 1.26 (1.01-1.57) |  | 200 | 1.05 (0.98-1.12) |  |
| **Education** |  |  |  |  |  |  |
| High school/GED or less | 134 | 1.49 (1.10-2.00) | 0.54 | 134 | 1.02 (0.95-1.10) | 0.75 |
| More than high school/GED | 266 | 1.21 (0.98-1.50) |  | 266 | 1.05 (0.98-1.12) |  |
| **Household income** |  |  |  |  |  |  |
| <$40,000 | 126 | 1.18 (0.89-1.57) | 0.52 | 126 | 1.03 (0.94-1.12) | 0.32 |
| **≥**$40,000 | 274 | 1.32 (1.07-1.62) |  | 274 | 1.07 (1.01-1.14) |  |
| **Years in the US** |  |  |  |  |  |  |
| <23 years | 200 | 1.57 (1.17-2.11) | 0.29 | 200 | 1.09 (1.01-1.17) | 0.37 |
| **≥**23 years | 200 | 1.21 (0.96-1.54) |  | 200 | 1.03 (0.96-1.10) |  |
| **Self-rated acculturation** |  |  |  |  |  |  |
| Very Asian | 242 | 1.30 (1.06-1.59) | 0.66 | 242 | 1.06 (1.00-1.12) | 0.32 |
| Mostly Asian | 62 | -^d^ |  | 62 | 1.09 (0.95-1.25) |  |
| Bicultural/mostly Westernized/very Westernized | 96 | 2.06 (1.09-3.90) |  | 96 | 1.01 (0.87-1.17) |  |
| **Self-rated English proficiency** |  |  |  |  |  |  |
| “Fluent like a native speaker” or “well” | 91 | 1.12 (0.74-1.69) | 0.22 | 91 | 1.12 (0.98-1.28) | 0.75 |
| “So-so” | 148 | 1.42 (0.99-2.04) |  | 148 | 1.06 (0.97-1.16) |  |
| “Poorly” or “not at all” | 161 | 1.50 (1.13-1.98) |  | 161 | 1.03 (0.97-1.10) |  |
| **Sleep apnea** |  |  |  |  |  |  |
| Low risk | 350 | 1.22 (1.01-1.46) | 0.25 | 350 | 1.05 (0.99-1.11) | 0.29 |
| High risk | 50 | -^d^ |  | 50 | 1.05 (0.93-1.18) |  |
| **Sleep disturbance** |  |  |  |  |  |  |
| None to slight | 372 | 1.17 (0.94-1.45) | 0.35 | 372 | 1.04 (0.98-1.11) | 0.56 |
| Mild/moderate/severe | 73 | 1.90 (1.15-3.14) |  | 73 | 1.04 (0.95-1.15) |  |
| **Sleep duration** |  |  |  |  |  |  |
| <6 hours | 39 | -^d^ | 0.19 | 39 | 1.25 (1.07-1.47) | 0.11 |
| **≥**6 hours | 351 | 1.23 (1.02-1.48) |  | 351 | 1.03 (0.98-1.09) |  |

^a^ Odds ratio (OR) and 95% confidence interval (CI) were estimated using logistic regression models adjusting for age, sex, Asian subgroup, marital status, education, household income, employment status, and health insurance status.

^b^ p-interaction was estimated using Wald test for interaction terms

^c^ Prevalence ratio (PR) and 95% confidence interval (CI) were estimated using Poisson regression models with a robust error variance, adjusting for age, sex, Asian subgroup, marital status, education, household income, employment status, and health insurance status.

^d^ Values were not estimated due to small case count within the stratum
